# Antifungal, Antileishmanial, and Cytotoxicity Activities of Various Extracts of *Berberis vulgaris* (Berberidaceae) and Its Active Principle Berberine

**DOI:** 10.1155/2014/602436

**Published:** 2014-03-10

**Authors:** Hossein Mahmoudvand, Seyyed Amin Ayatollahi Mousavi, Asghar Sepahvand, Fariba Sharififar, Behrouz Ezatpour, Fatemeh Gorohi, Ebrahim Saedi Dezaki, Sareh Jahanbakhsh

**Affiliations:** ^1^Leishmaniasis Research Center, Kerman University of Medical Sciences, Kerman 76169-14119, Iran; ^2^Razi Herbal Medicines Research Center, Lorestan University of Medical Sciences, Khorramabad 68138-33946, Iran; ^3^Department of Medical Parasitology and Mycology, Kerman University of Medical Sciences, Kerman 76169-14114, Iran; ^4^Department of Medical Parasitology and Mycology, Lorestan University of Medical Sciences, Khorramabad 68138-33946, Iran; ^5^Department of Pharmacognosy, Faculty of Pharmacy, Kerman University of Medical Sciences, Kerman 76169-14111, Iran; ^6^Department of Entomology, Islamic Azad University, Science and Research Branch, Tehran 14778-93855, Iran

## Abstract

In this study, *in vitro* antidermatophytic activity against *Trichophyton mentagrophytes*, *Trichophyton rubrum*, *Microsporum canis*, and *Microsporum gypseum* was studied by disk diffusion test and assessment of minimum inhibitory concentration (MIC) using CLSI broth macrodilution method (M38-A2). Moreover, antileishmanial and cytotoxicity activity of *B. vulgaris* and berberine against promastigotes of *Leishmania major* and *Leishmania tropica* were evaluated by colorimetric MTT assay. The findings indicated that the various extracts of *B. vulgaris* particularly berberine showed high potential antidermatophytic against pathogenic dermatophytes tested with MIC values varying from 0.125 to >4 mg/mL. The results revealed that *B. vulgaris* extracts as well as berberine were effective in inhibiting *L. major* and *L. tropica* promastigotes growth in a dose-dependent manner with IC_50_ (50% inhibitory concentration) values varying from 2.1 to 26.6 **μ**g/mL. Moreover, it could be observed that berberine as compared with *B. vulgaris* exhibited more cytotoxicity against murine macrophages with CC_50_ (cytotoxicity concentration for 50% of cells) values varying from 27.3 to 362.6 **μ**g/mL. Results of this investigation were the first step in the search for new antidermatophytic and antileishmanial drugs. However, further works are required to evaluate exact effect of these extracts in animal models as well as volunteer human subjects.

## 1. Introduction 

Dermatophytosis is a common contagious disease caused by a fungus known as dermatophytes. Dermatophytes belong to three genera of* Trichophyton*,* Microsporum*, and* Epidermophyton*, which are able to break down keratin in tissues such as epidermis, hair, nails, feathers, horns, and hooves. There are various forms of this disease including tinea corporis, tinea pedis, tinea capitis, tinea barbae, tinea cruris, tinea manuum, and onychomycosis [1]. At present, there are several antidermatophyte drugs such as imidazoles and terbinafine for topical treatment and triazoles, griseofulvin, and terbinafine as oral antifungal for systemic treatment of dermatophytosis which have shown some problems including high toxicity, fungal resistance, and prolonged systemic therapy [1–3]. These factors express urgent needs for development of new effective treatment alternatives. Leishmaniasis is caused by parasitic protozoa of the genus of* Leishmania* transmitted by bites of phlebotomine sand flies [4]. It is still considered a major health problem in tropical and subtropical countries worldwide and its clinical symptoms are varied, ranging from simple cutaneous lesion to fatal disease [4, 5]. In Iran, both epidemiological forms of cutaneous leishmaniasis (CL) are present, Anthroponotic CL (ACL) and zoonotic CL (ZCL) caused by* Leishmania tropica *and* Leishmania major*, respectively [5, 6]. First choice treatment drugs, which are antimonials such as meglumine antimoniate and sodium stibogluconate, have limitations for being used due to the prolonged duration of treatment, high toxicity, and high emergence of resistant parasites against these drugs [7–9]. These factors render development of new effective antileishmanial drugs as a requirement. Since a long time ago, medicinal plants and their compounds have been important and valuable sources for treatment and prevention of diseases [10]. Barberry,* Berberis vulgaris* (family Berberidaceae), grows in Asia and Europe.* B. vulgaris* called “*Zereshk*” in Persian is native to south-east of Iran. Different parts of this plant including root, leaf, bark, and fruit have been widely used as folk medicine for treatment and prevention of various diseases including cardiovascular, gastrointestinal, respiratory, skin, renal, and infectious ones [11]. Previous studies have been carried out on chemical composition of* B. vulgaris *and shown that the most important constituents of this plant are isoquinoline alkaloids such as berbamine, palmatine, and particularly berberine [12, 13]. So far, various studies have demonstrated antibacterial and antiparasitic effects of this plant against several pathogenic strains due to having berberine [14–17]. Moreover, antifungal effect of* B. vulgaris* and its main component, berberine, against* candida *spp. has been discovered in some investigations [18, 19]. In the present study, the* in vitro* antidermatophyte, antileishmanial, and cytotoxicity activities of various extracts of* B. vulgaris* and its active principle, berberine, were described against* Trichophyton mentagrophytes*,* Trichophyton rubrum*,* Microsporum canis*, and* Microsporum gypseum* as pathogenic dermatophyte strains and* Leishmania major* and* Leishmania tropica *as parasitic.

## 2. Materials and Methods

### 2.1. Chemicals

MTT powder [3-(4.5-dimethylthiazol-2-yl)-2.5-diphenyl tetrazolium bromide], fetal calf serum (FCS), and RPMI-1640 medium with L-glutamine and sabouraud dextrose broth (SDA) were purchased from Sigma-Aldrich, (St. Louis, MO, USA). Meglumine antimoniate (MA, Glucantime) as a control drug in the evaluation of antileishmanial effects was prepared from Rhône, Poulenc, France. Penicillin and streptomycin were obtained from Alborz Pharmacy, Karaj, Iran, and were stored at room temperature (25°C) until testing. Also, potato dextrose agar (PDA) was prepared from Oxoid, Basingstoke, Hampshire, United Kingdom. All the other chemicals and solvents were of analytical grade.

### 2.2. Dermatophyte Strains

Standard strains of* T. mentagrophytes* (PTCC 5054),* T. rubrum* (PTCC 5143),* M. canis* (PTCC 5069), and* M. gypseum* (PTCC 5070) were obtained from the center for Persian Type Culture Collection (Tehran, Iran) and were incubated in Sabouraud dextrose agar (SDA) at 30°C for 7−10 days.

### 2.3. Parasite Strains

Standard strains of* L. major* (MRHO/IR/75/ER) and* L. tropica* (MHOM/IR/2002/Mash2) were obtained from the Center for Research and Training in Skin Diseases and Leprosy (Tehran, Iran). The parasite was cultured in NNN medium, subcultured in RPMI-1640, supplemented with penicillin (200 IU/mL), streptomycin (100 *μ*g/mL), and 15% heat-inactivated fetal calf serum (FCS).

### 2.4. Preparation of Extracts

#### 2.4.1. Plant Collection and Authentication

The* B. vulgaris* root due to having the high percentage of berberine [11] was collected from the farms in the Baft district of Kerman province, Iran, in May 2012. The identities were confirmed by the botanist at the Botany Department of Shahid Bahonar University, Kerman, Iran. Voucher specimen (kf585) of the plant materials was deposited at the Herbarium of Department of Pharmacognosy of the School of Pharmacy, Kerman University of Medical Science, Kerman, Iran.

#### 2.4.2. Obtaining the Aqueous Extract

Fifty grams of plant material were ground and boiled gently with 500 mL distilled water for approximately 1 h. The filtered aqueous extracts were concentrated in a rotary vacuum evaporator and dried by exposure to hot air to yield solid material and then were stored at −20°C until testing.

#### 2.4.3. Preparation of the Methanolic and Chloroform Extracts

The dried plant materials (500 g) were grounded and extracted by percolation method by chloroform and methanol successively for 72 h in room temperature. Solvents were removed in a rotary evaporator and extracts were concentrated to dryness and stored at −20°C until testing. The* B. vulgaris* root yielded 29.2%, 25.1%, and 27.6% aqueous, chloroform, and methanolic extract of dried plant materials.

#### 2.4.4. Preparing of the Berberine

Berberine (2,3-methylenedioxy-9,10-dimethoxyprotoberberine chloride) as main compound of* B. vulgaris* was prepared from Sigma-Aldrich, (St. Louis, MO, USA) and dissolved in the dimethyl sulfoxide (DMSO). Final concentration of DMSO never exceeded 1% either in control or treated samples.

### 2.5. Preparing Peritoneal Macrophage Cells

In order to investigate cytotoxicity activity of* B. vulgaris *and berberine, peritoneal macrophages were collected from male healthy BALB/c mice (4–8 weeks old) by injecting 2–5 mL of cold RPMI-1640 medium into mouse peritoneal cavity. Then, the aspirated macrophages were washed twice and resuspended in RPMI-1640 medium. The experimental procedures carried out in this survey were in compliance with Standard Guidelines of Kerman University of Medical Sciences (Kerman, Iran) for Care and Use of Laboratory Animals.

In this investigation, antidermatophytic activities of various extracts of* B. vulgaris* and its main constituent, berberine, were evaluated by disk diffusion method and also minimum inhibitory concentration (MIC) was determined using broth macrodilution method.

### 2.6. Disk Diffusion Method

To assess antidermatophytic activity of* B. vulgaris *and berberine by disk diffusion method, fresh cultures of* T. mentagrophytes*,* T. rubrum*,* M. canis*, and* M. gypseum* after 3 weeks were spread on SDA. Then, filter paper disks (0.5 cm) were loaded with 1 mg/disk of various extracts of* B. vulgaris* (1 mg/disk) and berberine (0.01 mg/disk). Also, ketoconazole (0.01 mg/disk) and dimethyl sulfoxide (DMSO 1%), methanol, and water were used as positive and negative controls, respectively. After evaporation of the solvents, the disks were placed on the SDA plates. They were incubated at 30°C for 7–14 days and were measured for the developing diameter of inhibition zones around the disks. Finally, inhibition zone diameter was measured and the results were recorded. All the experiments were repeated in triplicate.

### 2.7. Determining Minimum Inhibitory Concentration (MIC)

The MIC determination of various extracts of* B. vulgaris* andberberine was carried out by broth macrodilution method, according to M38-A2 protocol of Clinical and Laboratory Standards Institute (CLSI) for filamentous fungi with some modifications [20]. At first, dermatophyte strains were subcultured on PDA slants and incubated at 30°C for 7 to 10 days. Mature colonies were covered with 2 mL of sterile physiological saline (0.85%), suspensions were prepared by gently probing the colony with the tip of a sterile Pasteur pipette and transferred to a sterile conical tube, and the final volumewas adjusted to 5 mL with saline. The resulting mixture of conidia and hyphal was vortex mixed for 15 sec and the heavy particles were allowed to settle for 5–10 min. The upper homogeneous suspension was used for further testing. The resulting suspension was counted in a Neubauer chamber and standardized to concentrations of 1 × 10^5^ to 5 × 10^5^ cfu/mL. This suspension was further diluted 1 : 10 with RPMI-1640 medium broth with L-glutamine and without sodium bicarbonate to final concentrations of 1 × 10^4^ to 5 × 10^4^ cfu/mL. For the broth macrodilution method, 0.9 mL of the final conidia suspensions was mixed with 0.1 mL of various concentrations of extracts of* B. vulgaris* (0.25–4 mg/mL) andberberine (0.062–0.5 mg/mL) in test tubes and incubated at 30°C for seven days. The positive control tube contained 0.9 mL of conidial suspension and 0.1 mL of RPMI-1640 and the negative one only contained 1 mL of RPMI-1640. The minimum concentrations at which no visible growth was observed were defined as MIC, which were expressed in mg/mL.

### 2.8. Antileishmanial Activity

Antileishmanial activity of* B. vulgaris* extracts and berberine was evaluated on promastigote stages of* L. major* and* L. tropica* by colorimetric cell viability (MTT) assay using the method described by Shokri et al. [21]. Briefly, 100 *μ*L of the promastigotes (10^6^ cells/mL) harvested from logarithmic growth phase was added to a 96-well microtiter plate. Then, 100 *μ*L of different concentrations (0–200 *μ*g/mL) of each plant extract was added to each well and incubated at 37°C ± 1°C for 72 h. After incubation with the extracts, 10 *μ*L of MTT solution (5 mg/mL) was added to each well and incubated at 37°C for 4 h. The promastigotes were cultured in the complete medium with no drug used as positive control and with no promastigotes and drugs as blank. Finally, absorbance was measured by an ELISA reader (BioTek-ELX800) at 490 nm. 50% inhibitory concentrations (IC_50_ values) were also calculated by Probit test in SPSS software.

### 2.9. Cytotoxicity Activity

In order to evaluate cytotoxic activity on peritoneal macrophage cells, CC_50_ (cytotoxicity concentration for 50% of cells) of various extracts of* B. vulgaris* and berberine on peritoneal murine macrophages were determined. The macrophage cells were plated at 10^6^ cells/mL in 96-well Lab-Tek (Nunc, USA) and left to adhere for 2 h at 37°C in 5% CO_2_. Nonadherent cells were removed by washing with medium after 2 h of incubation in similar conditions. In the next step, 190 *μ*L of complete RPMI medium was added to each well and later 10 *μ*L of dilutions of the extracts (previously prepared in medium) was added. The macrophages were treated with the extracts from 10 to 500 *μ*g/mL for 72 h [22]. The cytotoxicity was evaluated using the colorimetric assay with MTT as described previously in the promastigote assay.

### 2.10. Statistical Analyses

In this work, all the experiments were repeated in triplicate. SPSS software, Version 17, (SPSS Inc., Chicago) was used for data entry and statistical analysis and differences between the groups were determined using one-way Analysis of Variance (ANOVA) test. Moreover, to compare CC_50_ and IC_50_ values of the groups, *t*-test was performed. *P* value of less than 0.05 was considered statistically significant. The selectivity index (SI) calculated based on the equation of CC_50_ for peritoneal macrophage cells/IC_50_ for promastigote forms of both species of* Leishmania* was used to compare toxicity and activity of the crude extracts and berberine, as described by Weniger et al. [23].

## 3. Results

### 3.1. Antidermatophytic Activity

In evaluating antidermatophytic effects of various extracts of* B. vulgaris* andberberine by disk diffusion method, it could be observed that all the aforementioned extracts and berberine had potent antidermatophytic effects against* T. mentagrophytes*,* T. rubrum*,* M. canis*, and* M. gypseum* as pathogenic dermatophyte strains (Table 1). Berberine was much more effective than various extracts of* B. vulgaris* once it exhibited wider zone diameter of inhibition (>50 mm) for all the tested dermatophyte strains. Moreover, among the tested extracts of* B. vulgaris*, methanol extract showed higher antidermatophytic effects (from 26.3 to 37.6 mm) than chloroform (from 24.3 to 32 mm) and aqueous extracts while the lowest antidermatophytic effect was related to aqueous extract (from 18.3 to 25.3 mm) of* B. vulgaris*. The present results revealed that* M. canis* and* T. mentagrophytes* were the most sensitive and resistant strains to various extracts of* B. vulgaris* and berberine, respectively. It should be mentioned that negative controls were not shown any inhibition zone against the tested dermatophte strains.  Table 2 demonstrates results of MIC values of different extracts of* B. vulgaris* and berberine by broth macrodilution method. It could be seen that MIC values similar to disk diffusion method confirmed that berberine had more antidermatophytic effects than various extracts of* B. vulgaris* with the lowest MIC values for all the tested dermatophyte strains.

### 3.2. Antileishmanial Activity

In the investigation of antileishmanial effects of* B. vulgaris* extracts and berberine using MTT assay, it could be seen that various extracts of* B. vulgaris*, particularly berberine,significantly (*P* < 0.05) suppressed growth rate of promastigotes in both strains based on a dose-dependent response. Berberine indicated much higher activity against promastigote forms than crude extracts of* B. vulgaris*. Similarly, methanolic extract was more effective in inhibiting promastigotes growth than aqueous and chloroformextracts of* B. vulgaris*. Aqueous extract also revealed the lowest antileishmanial activity against these parasitic forms. The measured IC_50_ values forberberine were 2.1 *μ*g/mL and 2.9 *μ*g/mL against promastigote forms of* L. major* and* L. tropica*, respectively. While the measured IC_50_ values for various extracts of* B. vulgaris* and MA as control drug against promastigote forms of* L. major* and* L. tropica* were higher than berberine (Table 3).

### 3.3. Cytotoxicity Activity

Crude extracts of* B. vulgaris* showed no significant cytotoxicity on peritoneal macrophage cells while berberine as the active constituent displayed a more cytotoxicity effect at high concentrations ≥25 *μ*g/mL on peritoneal macrophages. The CC_50_ values for different extracts of* B. vulgaris* and also berberine against peritoneal macrophage cells and selectivity index (SI) are shown in Table 4.

## 4. Discussion

Since ancient times, plant-derived compounds and plant extracts have been used as a valuable natural resource of traditional remedy [24]. In the past decades, advent of synthetic antimicrobial drugs has caused reluctance in plants as a rich source of antimicrobial agents [25]. However, in recent years, emergence of some limitations in the use of these drugs has caused change in situation and interest in the field of ethnobotanical research [26]. In the present study, high potential* B. vulgaris* extracts and especially its active principle, berberine, were demonstrated as a natural source for the production of new antidermatophytic and antileishmanial drugs on an* in vitro* model. As mentioned, based on the previous studies, the highest biological activity of the* B. vulgaris* was due tohaving isoquinoline alkaloids such as berberine. This alkaloid is sparingly soluble in water and it is expected that the liquid extraction contains negligible amounts of berberine, whereas methanolic extract can draw this alkaloid from plant roots. In the case of antifungal effects of berberine, there are few studies that approve high potential of berberine against some pathogenic fungal strains. One such study was conducted by Freile et al. [14] indicating that berberine had potent antifungal effects against different* Candida* spp. with MIC values varying from 16 to 128 *μ*g/mL. Furthermore, Ghaderi and Maleki Nejad [18] showed that* B. vulgaris* extracts had significant anticandidal effects, which were more prominent for methanolic extracts. Also, these researchers reported that anticandidal activity of* B. vulgaris* might be due to the presence of several bioactive compounds, especially berberine.The mechanism underlying antifungal activities of berberine is not fully understood. However, Park et al. [27] exhibited that inhibitory effect of berberine on growth of* Candida* was probably due to inhibition of both sterol and cell wall biosyntheses in this fungus. Previous researches have reported that berberine has a synergistic effect in combination with amphotericin B in the treatment of candidiasis in mice, and it combined with fluconazole against clinical isolates of* Candida albicans* resistant to fluconazole [19, 28]. Therefore, all of these results are consistent with results of the present study.

So far, in various investigations, antiparasitic effects of* B. vulgaris* and its bioactive compounds (particularly berberine) against some pathogenic parasite strains have been reported. The study conducted by Kaneda et al. [16] showed that berberine significantly reduced growth of* Entamoeba histolytica*,* Giardia lamblia*, and* Trichomonas vaginalis* on an* in vitro* model and caused morphological changes in their structure. Also, Sheng et al. [29] reported that, in chloroquine resistant malaria, the combination of berberine and pyrimethamine had a synergistic effect in the elimination of parasites and it was more effective than other drugs such as tetracycline or cotrimoxazole. In another survey, Vennerstrom et al. [17] demonstrated that berberine derivatives significantly suppressed the parasite load in liver or ulcer size in golden hamsters infected with* Leishmania donovani* and* Leishmania braziliensis* when compared with meglumine antimoniate. Fata et al. [15] also found that ethanolic extract of* B. vulgaris* significantly decreased ulcer size of cutaneous leishmaniasis of BALB/c mice after 2 weeks. Recently, Rouhani et al. [30] reported that aqueous extract of* B. vulgaris* fruits had an effective scolicidal activity at low concentration (4 mg/mL) and short exposure time (5 min). Thus, the present results were in agreement with those of previous studies in demonstrating antiparasitic activities of* B. vulgaris* and its bioactive compound, berberine.

In this survey, it was indicated that various extractsof* B. vulgaris* had no significant cytotoxicity effect at low concentrations in peritoneal macrophage cells while berberine indicated moderate cytotoxicity effects on peritoneal macrophage cells. Similar to these findings, Peychev [31] revealed that oral administration of* B. vulgaris* was moderately toxic in mice (LD_50_ = 2.6 ± 0.22 g/kg b.w). In contrast, Lin et al. [32] showed that berberine had strong inhibitory effect on proliferation of both hepatoma and leukemia cell lines on* in vitro* model. However, it has been proven that berberine is not considered toxic at the doses used in clinical situations nor has it been shown to be cytotoxic or mutagenic; however, some side-effects have been mentioned to result from high dosages [11]. In addition, SI_s_ ≥ 10 of extracts showed their safety to the macrophages and specificity to the parasite [23]. Thus, it could be proposed that* B. vulgaris* extracts were safe for mammalian cells considering that at high concentrations, they showed significant cytotoxicity in the host cells.

## 5. Conclusion 

To conclude, findings of this study could provide the first step in the search for new antidermatophytic and antileishmanial drugs and aid the use of* B. vulgaris* in folk medicine for dermatophytic and leishmaniasis infections. In addition, further studies are required to elucidate the exact effects of these extracts and berberine in animal models as well as volunteer human subjects as a new therapeutic agent.

## Figures and Tables

**Table 1:** Antidermatophytic effects of various extracts of *B. vulgaris* and its main constituent, berberine, against* T. mentagrophytes*,* T. rubrum*, *M. canis,* and *M. gypseum* by disk diffusion method. Data are expressed as the mean ± SD (*n* = 3).

Dermatophytes	Zone of inhibition (mm)				
	Chloroform extract	Methanolic extract	Aqueous extract	Berberine	Ketoconazole
*T. mentagrophytes *	24.3 ± 2.15	26.3 ± 1.52	18.3 ± 1.17	51.6 ± 3.08	28.6 ± 1.17
*T. rubrum *	25.3 ± 2.15	28.6 ± 2.08	21.6 ± 2.15	55 ± 3.08	30.3 ± 2.52
*M. canis *	32 ± 2.5	37.6 ± 3.08	25.3 ± 2.52	>60	37.6 ± 1.52
*M. gypseum *	27.6 ± 1.17	30.6 ± 1.52	23.3 ± 1.17	>60	31.6 ± 1.52

**Table 2:** Minimum inhibitory concentration (MIC) of various extractsof *B. vulgaris* and its main component, berberine, against* T. mentagrophytes*, *T. rubrum, M. canis,* and *M. gypseum* by broth macrodillution method.

Dermatophytes	MIC (mg/mL)				
	Chloroform extract	Methanolic extract	Aqueous extract	Berberine	Ketoconazole
*T. mentagrophytes *	4	4	>4	0.250	0.250
*T. rubrum *	4	2	4	0.125	0.250
*M. canis *	2	1	2	0.062	0.125
*M. gypseum *	4	1	4	0.125	0.250

**Table 3:** IC_50_ values of various extracts of *B. vulgaris* and berberine against the growth rate of promastigote forms of *Leishmania major* and* Leishmania tropica*. Data are expressed as the mean ± SD (*n* = 3).

Sample	IC_50_ value (µg/mL)^a^	
	*L. major *	*L. tropica *
Methanolic extract	12.8 ± 1.17	16.1 ± 1.15
Aqueous extract	22.3 ± 2.51	26.6 ± 2.51
Chloroform extract	19.6 ± 2.08	23.3 ± 2.15
Berberine	2.1 ± 0.05	2.9 ± 0.05
MA^b^	8.2 ± 1.15	11.6 ± 0.05

^a^Concentration of drug that caused 50% of growth inhibition of promastigotes.

^b^Meglumine antimoniate (Glucantime) as control drug.

**Table 4:** CC_50_ values of methanolic and aqueous extracts of *B. vulgaris* and berberine on peritoneal macrophage cells and selectivity index (SI) against promastigote forms of *L. major* and *L. tropica. *Data are expressed as the mean ± SD (*n* = 3).

Samples	CC_50_^a^ ± SD (*μ*g/mL)	SI^b^	
		*L. major *	*L. tropica *
Methanolic extract	203.3 ± 3.08	15.9	12.6
Aqueous extract	362.6 ± 4.6	16.3	13.8
Chloroform extract	297.6 ± 4.6	15.2	12.8
Berberine	27.3 ± 2.08	13	9.4

^a^Concentration of extracts that caused 50% mortality in BALB/c mice peritoneal macrophages.

^b^Selectivity index (CC_50_/IC_50_).
